# Supplementary material for: Prognostic Inflammatory Index Based on Preoperative Peripheral Blood for Predicting the Prognosis of Colorectal Cancer Patients
Source: Cancers (Basel). 2020 Dec 22;13(1):3. doi: 10.3390/cancers13010003 (PMC7792597; doi:10.3390/cancers13010003)
Supplement: Supplementary file 1 [file cancers-13-00003-s001.pdf]

Table S1. The prognostic effects of six types of preoperative blood cell count for patients with colorectal cancer in the training cohort.

Variables	Univariate Analysis [a]			Multivariate Analysis [b]		
	β	HR (95% CI)	*p* Value	β	HR (95% CI)	*p* Value
Overall Survival						
Platelet (10^9/L)						
>178	-	1.000	-	-	1.000	-
≤178	0.1514	1.163 (0.996–1.359)	0.057	0.1878	1.207 (1.029–1.414)	0.020
Lymphocyte (10^9/L)						
>1.34	-	1.000	-	-	1.000	-
≤1.34	0.1846	1.203 (1.047–1.381)	0.009	0.1370	1.147 (0.993–1.324)	0.062
Neutrophil (10^9/L)	0.0508	1.052 (1.023–1.083)	<0.001	0.0251	1.025 (0.992–1.060)	0.138
Monocyte (10^9/L)	0.5172	1.677 (1.294–2.174)	<0.001	0.4570	1.579 (1.175–2.124)	0.002
Eosinophil (10^9/L)						
>0.04	-	1.000	-	-	1.000	-
≤0.04	0.2451	1.278 (1.114–1.466)	<0.001	0.2094	1.233 (1.070–1.421)	0.004
Basophil (10^9/L)	−0.6651	0.514 (0.085–3.104)	0.468	-	-	-
Disease-free Survival						
Platelet (10^9/L)						
>185	-	1.00	-	-	1.00	-
≤185	0.1956	1.216 (1.039–1.424)	0.015	0.2370	1.267 (1.079–1.489)	0.004
Lymphocyte (10^9/L)	−0.0191	0.981 (0.896–1.075)	0.682	-	-	-
Neutrophil (10^9/L)	0.0480	1.049(1.016–1.083)	0.003	0.0415	1.042 (1.004–1.082)	0.028
Monocyte (10^9/L)	0.3414	1.407 (1.042–1.900)	0.026	0.2600	1.297 (0.917–1.835)	0.142
Eosinophil (10^9/L)						
>0.11	-	1.00	-	-	1.00	-
≤0.11	0.2393	1.270 (1.123–1.437)	<0.001	0.2437	1.276 (1.127–1.445)	<0.001
Basophil (10^9/L)	-0.5663	0.568 (0.075–4.318)	0.584	-	-	-

[a] Univariate analyses were performed to investigate the association between the six types of blood cell counts and overall survival or disease-free survival. Blood cells whose statistical $p < 0.10$ was defined as that associated with prognosis of colorectal cancer. [b] Significant prognostic factors ($p < 0.10$) in the univariate analyses were entered into multivariate models. The HRs were obtained based on the mutual adjustment of different factors. Coefficients of the multivariate Cox model were used as the weights of blood cell counts for the construction of the prognostic inflammatory index. Abbreviations: HR, hazard ratio; CI, confidence interval.

Table S2. Association of OS-PII and DFS-PII with clinicopathological characteristics of colorectal cancer in the training cohort.

Demographic or Characteristic	Case (%)	OS-PII		*p* Value	DFS-PII		*p* Value
		≤4.27	>4.27		≤4.47	>4.47	
Age (year)				0.001			0.174
<60	2103 (50.6)	1411 (52.5)	692 (47.2)		1080 (51.7)	1023 (49.5)	
≥60	2051 (49.4)	1278 (47.5)	773 (52.8)		1009 (48.3)	1042 (50.5)	
Gender				<0.001			0.920
Male	2454 (59.1)	1522 (56.6)	932 (63.6)		1232 (59.0)	1222 (59.2)	
Female	1700 (40.9)	1167 (43.4)	533 (36.4)		857 (41.0)	843 (40.8)	
BMI (kg/m^2)				0.016			0.201
<24	2390 (57.5)	1516 (56.4)	874 (59.7)		1188 (56.9)	1202 (58.2)	
≥24	1764 (42.5)	1173 (43.6)	591 (40.3)		901 (43.1)	863 (41.8)	
Hypertension				0.033			0.139
No	3554 (85.6)	2277 (84.7)	1277 (87.2)		1770 (84.7)	1784 (86.4)	
Yes	600 (14.4)	412 (15.3)	188 (12.8)		319 (15.3)	281 (13.6)	
Diabetes mellitus				0.097			0.323
No	3842 (92.5)	2501 (93.0)	1341 (91.5)		1941 (92.9)	1901 (92.1)	
Yes	312 (7.5)	188 (7.0)	124 (8.5)		148 (7.1)	164 (7.9)	
Tumor location				0.001			0.548
Colon	1961 (47.2)	1219 (45.3)	742 (50.6)		976 (46.7)	985 (47.7)	

Rectum	2193 (52.8)	1470 (54.7)	723 (49.4)		1113 (53.3)	1080 (52.3)	
Tumor diameter				0.034			0.382
<50 mm	1696 (40.8)	1134 (42.2)	563 (38.4)		871 (41.7)	825 (40.0)	
≥50 mm	2458 (59.2)	1555 (57.8)	902 (61.6)		1218 (58.3)	1240 (60.0)	
Pathological classification				0.492			0.871
Prominence	2740 (66.0)	1791 (66.6)	949 (64.8)		1377 (65.9)	1363 (66.0)	
Infiltration or Ulceration	428 (10.3)	271 (10.1)	157 (10.7)		211 (10.1)	217 (10.5)	
Infiltration and Ulceration	986 (23.7)	627 (23.3)	359 (24.5)		501 (24.0)	485 (23.5)	
Differentiation degree				<0.001			0.012
Well	331 (8.0)	226 (8.4)	105 (7.2)		175 (8.4)	156 (7.6)	
Moderate	3225 (77.6)	2124 (79.0)	1101 (75.2)		1646 (78.8)	1579 (76.5)	
Poor	598 (14.4)	339 (12.6)	259 (17.6)		268 (12.8)	330 (15.9)	
Histologic classification				0.851			0.281
Adenocarcinoma	3136 (75.5)	2033 (75.6)	1103 (75.3)		1592 (76.2)	1544 (74.8)	
Mucinous adenocarcinoma or signet ring cell carcinoma	1018 (24.5)	656 (24.4)	362 (24.7)		497 (23.8)	521 (25.2)	
TNM staging				0.062			1.000
II	2385 (57.4)	1515 (56.3)	870 (59.4)		1199 (57.4)	1186 (57.4)	
III	1769 (42.6)	1174 (43.7)	595 (40.6)		890 (42.6)	879 (42.6)	
Tumor invasion				0.011			0.073
T1–T3	1847 (44.5)	1235 (45.9)	612 (41.8)		958 (45.9)	889 (43.1)	
T4	2307 (55.5)	1454 (54.1)	853 (58.2)		1131 (54.1)	1176 (56.9)	
Cancer nodules				0.436			0.730
No	3863 (93.0)	2449 (92.8)	1404 (93.5)		1946 (93.2)	1917 (92.8)	
Yes	291 (7.0)	191 (7.2)	98 (6.5)		143 (6.8)	148 (7.2)	
Nerve invasion				0.840			0.224
No	3836 (92.3)	2481 (92.3)	1355 (92.5)		1940 (92.9)	1896 (91.8)	
Yes	318 (7.7)	208 (7.7)	110 (7.5)		149 (7.1)	169 (8.2)	
Vascular tumor thrombus				0.440			0.810
No	4009 (96.5)	2600 (96.7)	1409 (96.2)		2018 (96.6)	1991 (96.4)	
Yes	145 (3.5)	89 (3.3)	56 (3.8)		71 (3.4)	74 (3.6)	
CEA				0.377			0.947
<5 ng/mL	2438 (58.7)	1590 (59.1)	848 (57.9)		1231 (58.9)	1207 (58.5)	
≥5 ng/mL	1716 (41.3)	1099 (40.9)	617 (42.1)		858 (41.1)	858 (41.5)	
CA19-9				<0.001			0.001
<37 U/mL	3452 (83.1)	2278 (84.7)	1174 (80.1)		1773 (85.9)	1679 (81.3)	
≥37 U/mL	702 (16.9)	411 (15.3)	291 (19.9)		316 (14.1)	386 (18.7)	

Abbreviations: BMI, body mass index; TNM, Tumor-node-metastasis.

Table S3. Association of OS-PII and DFS-PII with clinicopathological characteristics of colorectal cancer in the validation cohort.

Demographic or Characteristic	Case (%)	OS-PII		*p* Value	DFS-PII		*p* Value
		≤4.27	>4.27		≤4.47	>4.47	
Age (year)				<0.001			0.005
<60	2645 (51.2)	1886 (53.8)	759 (45.8)		1530 (53.0)	1115 (49.0)	
≥60	2516 (48.8)	1618 (46.2)	898 (54.2)		1356 (47.0)	1160 (51.0)	
Gender				<0.001			0.101
Male	3062 (59.3)	1962 (56.0)	1100 (66.4)		1683 (58.3)	1379 (60.6)	
Female	2099 (40.7)	1542 (44.0)	557 (33.6)		1203 (41.7)	896 (39.4)	
Tumor location				0.012			0.429
Colon	2465 (47.8)	1631 (46.5)	834 (50.3)		1393 (48.3)	1072 (47.1)	
Rectum	2696 (52.2)	1873 (53.5)	823 (49.7)		1493 (51.7)	1203 (52.9)	
Tumor diameter				0.001			0.320
<50 mm	3450 (66.8)	2395 (68.4)	1055 (63.7)		1912 (66.3)	1538 (67.6)	
≥50 mm	1711 (33.2)	1109 (31.6)	602 (36.3)		974 (33.7)	737(32.4)	
Pathological classification				0.140			0.377
Prominence	1277 (24.7)	853 (24.3)	424 (25.7)		699 (24.2)	578 (25.4)	

Infiltration or Ulceration	3674 (71.2)	2519 (71.9)	1155 (69.6)		2075 (71.9)	1599 (70.3)	
Infiltration and Ulceration	210 (4.1)	132 (3.8)	78 (4.7)		112 (3.9)	98 (4.3)	
Differentiation degree				0.003			0.094
Well	48 (0.9)	37 (1.1)	11 (0.7)		20 (0.7)	28 (1.2)	
Moderate	3755 (72.8)	2593 (74.0)	1162 (70.1)		2129 (73.8)	1626 (71.5)	
Poor	1358 (26.3)	874 (24.9)	484 (29.2)		737 (25.5)	621 (27.3)	
Histologic classification				0.138			0.121
Adenocarcinoma	4342 (84.1)	2965 (84.6)	1377 (83.1)		2448 (84.8)	1894 (83.3)	
Mucinous adenocarcinoma or signet ring cell carcinoma	819 (15.9)	539 (15.4)	280 (16.9)		438 (15.2)	381 (16.7)	
TNM staging				0.513			0.086
II	2086 (40.4)	1405 (40.1)	681 (41.1)		1197 (41.5)	889 (39.1)	
III	3075 (59.6)	2099 (59.9)	976 (58.9)		1689 (58.5)	1386 (60.9)	
Tumor invasion				0.272			0.156
T1–T3	2588 (50.1)	1776 (50.7)	812 (49.0)		1473 (51.0)	1115 (49.0)	
T4	2573 (49.9)	1728 (49.3)	845 (51.0)		1413 (49.0)	1160 (51.0)	
Cancer nodules				0.106			0.359
No	4249 (82.3)	2906 (82.9)	1343 (81.1)		2389 (82.8)	1860 (81.8)	
Yes	912 (17.7)	598 (17.1)	314 (18.9)		497 (17.2)	415 (18.2)	
Nerve invasion				0.735			0.035
No	3898 (75.5)	2641 (75.4)	1257 (75.9)		2213 (76.7)	1685 (74.1)	
Yes	1263 (24.5)	863 (24.6)	400 (24.1)		673 (23.3)	590 (25.9)	
Vascular tumor thrombus				0.736			0.031
No	3622 (70.2)	2465 (70.3)	1157 (69.8)		2060 (71.4)	1562 (68.7)	
Yes	1539 (29.8)	1039 (29.7)	500 (30.2)		826 (28.6)	713 (31.3)	
CEA				<0.001			0.088
<5 ng/mL	2993 (58.0)	2096 (59.8)	897 (54.1)		1703 (59.0)	1290 (56.7)	
≥5 ng/mL	2168 (42.0)	1408 (40.2)	760 (45.9)		1183 (41.0)	985 (43.3)	
CA19-9				0.001			0.026
<37 U/mL	4137 (80.2)	2857 (81.5)	1280 (77.2)		2348 (81.4)	1789 (78.6)	
≥37 U/mL	1024 (19.8)	647 (18.5)	377 (22.8)		538 (18.6)	486 (21.4)	

Abbreviations: BMI, body mass index; TNM, Tumor-node-metastasis.

Table S4. The overall survival rates at 1-, 3-, 5- and 10-year in groups stratified by OS-PII in the training cohort.

Group	1-Year		3-Year		5-Year		10-Year	
	**OSR (SE) [a]**	***p* Value**	**OSR (SE) [a]**	***p* Value**	**OSR (SE) [a]**	***p* Value**	**OSR (SE) [a]**	***p* Value**
All patients (*N* = 4154)	0.946 (0.004)		0.808 (0.006)		0.723 (0.007)		0.631 (0.010)	
OS-PII		<0.001		<0.001		<0.001		<0.001
≤4.27 (*N* = 2689)	0.956 (0.004)		0.833 (0.007)		0.747 (0.009)		0.672 (0.012)	
>4.27 (*N* = 1465)	0.927 (0.007)		0.761 (0.011)		0.681 (0.012)		0.560 (0.018)	
Stage II patients (*N* = 2385)	0.975 (0.003)		0.898 (0.006)		0.818 (0.008)		0.712 (0.012)	
OS-PII		0.039		<0.001		0.002		<0.001
≤4.27 (*N* =1515)	0.980 (0.004)		0.920 (0.007)		0.837 (0.010)		0.752 (0.014)	
>4.27 (*N* =870)	0.966 (0.006)		0.858 (0.012)		0.785 (0.014)		0.644 (0.021)	
Stage III patients (*N* = 1769)	0.906 (0.007)		0.684 (0.011)		0.590 (0.012)		0.522 (0.024)	
OS-PII		<0.001		<0.001		<0.001		<0.001
≤4.27 (*N* =1174)	0.925 (0.008)		0.719 (0.013)		0.625 (0.015)		0.576 (0.020)	
>4.27 (*N* =595)	0.870 (0.014)		0.615 (0.020)		0.520 (0.021)		0.425 (0.046)	

[a] Overall survival rate (standard error)

Table S5. The disease-free survival rates at 1-, 3-, 5- and 10-year in groups stratified by DFS-PII in the training cohort.

Group	1-Year		3-Year		5-Year		10-Year	
	**DFSR (SE) [a]**	***p* Value**	**DFSR (SE) [a]**	***p* Value**	**DFSR (SE) [a]**	***p* Value**	**DFSR (SE) [a]**	***p* Value**
All patients (*N* = 4154)	0.920 (0.004)		0.810 (0.006)		0.759 (0.007)		0.715 (0.008)	
DFS-PII		<0.001		0.001		<0.001		<0.001

≤4.47 (*N* = 2089)	0.937 (0.005)		0.831 (0.008)		0.791 (0.009)		0.756 (0.012)	
>4.47 (*N* = 2065)	0.903 (0.007)		0.787 (0.009)		0.726 (0.010)		0.672 (0.013)	
Stage II patients (*N* = 2385)	0.958 (0.004)		0.885 (0.007)		0.842 (0.008)		0.809 (0.009)	
DFS-PII		<0.001		0.002		<0.001		<0.001
≤4.47 (*N* =1199)	0.975 (0.005)		0.906 (0.009)		0.871 (0.010)		0.843 (0.012)	
>4.47 (*N* =1186)	0.941 (0.007)		0.864 (0.010)		0.813 (0.012)		0.775 (0.013)	
Stage III patients (*N* = 1769)	0.869 (0.008)		0.702 (0.011)		0.637 (0.012)		0.526 (0.027)	
DFS-PII		0.051		0.079		0.010		0.003
≤4.47 (*N* =890)	0.886 (0.011)		0.727 (0.015)		0.677 (0.017)		0.604 (0.033)	
>4.47 (*N* =879)	0.851 (0.012)		0.676 (0.017)		0.594 (0.018)		0.443 (0.043)	

[a] Disease-free survival rate (standard error)

Table S6. The overall survival rates at 1-, 3-, 5-year in groups stratified by OS-PII in the validation cohort.

Group	1-Year		3-Year		5-Year	
	OSR (SE) [a]	*p* Value	OSR (SE) [a]	*p* Value	OSR (SE) [a]	*p* Value
All patients (*N* = 5161)	0.960 (0.003)		0.865 (0.006)		0.798 (0.010)	
OS-PII		<0.001		<0.001		<0.001
≤4.27 (*N* = 3504)	0.969 (0.003)		0.881 (0.007)		0.820 (0.011)	
>4.27 (*N* = 1657)	0.940 (0.006)		0.831 (0.012)		0.750 (0.019)	
Stage II patients (*N* = 2086)	0.987 (0.003)		0.939 (0.007)		0.884 (0.013)	
OS-PII		0.002		<0.001		<0.001
≤4.27 (*N* =1405)	0.993 (0.002)		0.956 (0.008)		0.918 (0.013)	
>4.27 (*N* =681)	0.974 (0.007)		0.907 (0.015)		0.813 (0.028)	
Stage III patients (*N* = 3075)	0.941 (0.005)		0.815 (0.009)		0.740 (0.013)	
OS-PII		<0.001		<0.001		0.001
≤4.27 (*N* =2099)	0.953 (0.005)		0.832 (0.011)		0.756 (0.017)	
>4.27 (*N* =976)	0.916 (0.009)		0.778 (0.017)		0.706 (0.024)	

[a] Overall survival rate (standard error)

Table S7. The disease-free survival rates at 1-, 3-, 5-year in groups stratified by DFS-PII in the validation cohort.

Group	1-Year		3-Year		5-Year	
	DFSR (SE) [a]	*p* Value	DFSR (SE) [a]	*p* Value	DFSR (SE) [a]	*p* Value
All patients (*N* = 5161)	0.901 (0.004)		0.749 (0.008)		0.689 (0.010)	
DFS-PII		0.003		0.002		<0.001
≤4.47 (*N* = 2886)	0.912 (0.006)		0.766 (0.010)		0.715 (0.013)	
>4.47 (*N* = 2275)	0.887 (0.007)		0.728 (0.012)		0.655 (0.016)	
Stage II patients (*N* = 2086)	0.951 (0.005)		0.855 (0.010)		0.802 (0.014)	
DFS-PII		0.004		0.005		0.002
≤4.47 (*N* =1197)	0.964 (0.006)		0.873 (0.013)		0.830 (0.017)	
>4.47 (*N* =889)	0.935 (0.009)		0.830 (0.016)		0.762 (0.023)	
Stage III patients (*N* = 3075)	0.867 (0.007)		0.679 (0.011)		0.614 (0.013)	
DFS-PII		0.105		0.093		0.048
≤4.47 (*N* =1689)	0.875 (0.009)		0.692 (0.014)		0.635 (0.018)	
>4.47 (*N* =1386)	0.856 (0.010)		0.663 (0.016)		0.589 (0.020)	

[a] Disease-free survival rate (standard error)

Table S8. Time-dependent ROC analyses for predicting overall survival of patients with colorectal cancer in the training cohort.

AUROC	NLR	PLR	LMR	SII	OS-PII	TNM	TNM + OS-PII	*p* Value [a]	*p* Value [b]	*p* Value [c]	*p* Value [d]	*p* Value [e]
1-year	0.581	0.578	0.587	0.596	0.586	0.671	0.713	0.832	0.705	0.787	0.957	<0.001
2-year	0.549	0.532	0.553	0.552	0.564	0.675	0.707	0.261	0.433	0.082	0.340	<0.001
3-year	0.542	0.519	0.543	0.538	0.562	0.676	0.709	0.085	0.072	0.006	0.066	<0.001
4-year	0.531	0.511	0.537	0.524	0.550	0.667	0.693	0.072	0.032	0.006	0.167	<0.001
5-year	0.524	0.502	0.529	0.515	0.545	0.665	0.687	0.040	0.012	0.002	0.091	<0.001
6-year	0.525	0.479	0.535	0.502	0.557	0.672	0.700	0.003	<0.001	<0.001	0.021	<0.001
7-year	0.525	0.469	0.536	0.498	0.566	0.694	0.727	<0.001	<0.001	<0.001	0.003	<0.001

8-year	0.514	0.463	0.520	0.489	0.547	0.706	0.732	0.009	<0.001	<0.001	0.021	<0.001
9-year	0.494	0.455	0.511	0.473	0.534	0.715	0.737	0.006	<0.001	<0.001	0.098	0.011
10-year	0.462	0.430	0.510	0.446	0.485	0.710	0.708	0.229	0.083	0.034	0.193	0.895
11-year	0.460	0.372	0.509	0.403	0.527	0.729	0.751	0.026	<0.001	<0.001	0.405	0.157

Abbreviations: AUROC, area under the receiver operating characteristic curve; TNM, Tumor-node-metastasis. The AUROC represented the prognostic prediction ability of Cox models involving single parameter, like NLR (continuous variable), PLR (continuous variable), LMR (continuous variable), SII (continuous variable), OS-PII (continuous variable), TNM (stage II and III), and combination of OS-PII and TNM. [a] Comparison of the AUROCs of OS-PII and NLR. [b] Comparison of the AUROCs of OS-PII and SII. [C] Comparison of the AUROCs of OS-PII and PLR. [d] Comparison of the AUROCs of OS-PII and LMR. [e] Comparison of the AUROCs of TNM and a combination of TNM and OS-PII.

Table S9. Time-dependent ROC analyses for predicting disease-free survival of patients with colorectal cancer in the training cohort.

AUROC	NLR	PLR	LMR	SII	DFS-PII	TNM	TNM + DFS-PII	*p* Value [a]	*p* Value [b]	*p* Value [c]	*p* Value [d]	*p* Value [e]
1-year	0.531	0.514	0.537	0.529	0.569	0.654	0.688	0.077	0.101	0.046	0.196	<0.001
2-year	0.520	0.503	0.522	0.517	0.549	0.656	0.680	0.064	0.077	0.021	0.124	<0.001
3-year	0.521	0.495	0.521	0.515	0.548	0.658	0.682	0.066	0.038	0.003	0.082	<0.001
4-year	0.519	0.490	0.521	0.508	0.550	0.661	0.686	0.025	0.005	<0.001	0.043	<0.001
5-year	0.522	0.486	0.525	0.506	0.558	0.668	0.697	0.006	<0.001	<0.001	0.016	<0.001
6-year	0.519	0.462	0.525	0.491	0.569	0.680	0.714	<0.001	<0.001	<0.001	0.002	<0.001
7-year	0.522	0.456	0.526	0.490	0.578	0.711	0.749	<0.001	<0.001	<0.001	0.001	<0.001
8-year	0.506	0.445	0.508	0.480	0.571	0.730	0.765	<0.001	<0.001	<0.001	<0.001	<0.001
9-year	0.489	0.434	0.495	0.464	0.562	0.751	0.781	<0.001	<0.001	<0.001	<0.001	<0.001
10-year	0.447	0.399	0.482	0.425	0.520	0.753	0.764	0.001	<0.001	<0.001	0.137	0.241
11-year	0.449	0.346	0.482	0.385	0.550	0.776	0.800	0.001	<0.001	<0.001	0.636	0.060

Abbreviations: AUROC, area under the receiver operating characteristic curve; TNM, Tumor-node-metastasis. The AUROC represented the prognostic prediction ability of Cox models involving single parameter, like NLR (continuous variable), PLR (continuous variable), LMR (continuous variable), SII (continuous variable), OS-PII (continuous variable), TNM (stage II and III), and combination of OS-PII and TNM. [a] Comparison of the AUROCs of DFS-PII and NLR. [b] Comparison of the AUROCs of DFS-PII and SII. [C] Comparison of the AUROCs of DFS-PII and PLR. [d] Comparison of the AUROCs of DFS-PII and LMR. [e] Comparison of the AUROCs of TNM and a combination of TNM and DFS-PII.

Table S10. Time-dependent ROC analyses for predicting overall survival of patients with colorectal cancer in the validation cohort.

AUROC	NLR	PLR	LMR	SII	OS-PII	TNM	TNM + OS-PII	*p* Value [a]	*p* Value [b]	*p* Value [c]	*p* Value [d]	*p* Value [e]
1-year	0.661	0.589	0.658	0.653	0.625	0.638	0.707	0.106	0.263	0.193	0.082	<0.001
2-year	0.623	0.554	0.603	0.602	0.599	0.645	0.702	0.155	0.861	0.050	0.794	<0.001
3-year	0.592	0.550	0.560	0.576	0.566	0.624	0.660	0.104	0.618	0.469	0.692	<0.001
4-year	0.592	0.520	0.539	0.551	0.575	0.613	0.654	0.283	0.257	0.019	0.014	<0.001
5-year	0.600	0.528	0.528	0.555	0.561	0.610	0.642	0.053	0.777	0.198	0.060	0.003
6-year	0.612	0.532	0.549	0.558	0.587	0.607	0.659	0.297	0.296	0.086	0.073	<0.001
7-year	0.669	0.608	0.638	0.633	0.628	0.579	0.662	0.305	0.916	0.696	0.794	<0.001

Abbreviations: AUROC, area under the receiver operating characteristic curve; TNM, Tumor-node-metastasis. The AUROC represented the prognostic prediction ability of Cox models involving single parameter, like NLR (continuous variable), PLR (continuous variable), LMR (continuous variable), SII (continuous variable), OS-PII (continuous variable), TNM (stage II and III), and combination of OS-PII and TNM. [a] Comparison of the AUROCs of OS-PII and NLR. [b] Comparison of the AUROCs of OS-PII and SII. [C] Comparison of the AUROCs of OS-PII and PLR. [d] Comparison of the AUROCs of OS-PII and LMR. [e] Comparison of the AUROCs of TNM and a combination of TNM and OS-PII.

Table S11. Time-dependent ROC analyses for predicting disease-free survival of patients with colorectal cancer in the validation cohort.

AUROC	NLR	PLR	LMR	SII	DFS-PII	TNM	TNM + DFS-PII	*p* Value[a]	*p* Value[b]	*p* Value[c]	*p* Value[d]	*p* Value [e]
1-year	0.587	0.552	0.587	0.577	0.550	0.613	0.637	0.043	0.194	0.920	0.528	0.002
2-year	0.558	0.521	0.544	0.540	0.549	0.622	0.647	0.531	0.601	0.128	0.737	<0.001
3-year	0.559	0.520	0.514	0.536	0.535	0.607	0.622	0.106	0.953	0.445	0.190	0.028

4-year	0.573	0.510	0.500	0.534	0.540	0.603	0.620	0.051	0.727	0.154	0.024	0.024
5-year	0.586	0.509	0.491	0.537	0.541	0.606	0.619	0.024	0.862	0.211	0.022	0.173
6-year	0.591	0.519	0.520	0.541	0.556	0.599	0.618	0.210	0.629	0.296	0.229	0.142
7-year	0.648	0.607	0.618	0.620	0.519	0.570	0.588	0.005	0.054	0.132	0.058	0.899

Abbreviations: AUROC, area under the receiver operating characteristic curve; TNM, Tumor-node-metastasis. The AUROC represented the prognostic prediction ability of Cox models involving single parameter, like NLR (continuous variable), PLR (continuous variable), LMR (continuous variable), SII (continuous variable), OS-PII (continuous variable), TNM (stage II and III), and combination of OS-PII and TNM. [a] Comparison of the AUROCs of DFS-PII and NLR. b Comparison of the AUROCs of DFS-PII and SII. C Comparison of the AUROCs of DFS-PII and PLR. d Comparison of the AUROCs of DFS-PII and LMR. e Comparison of the prognostic accuracy of TNM and a combination of TNM and DFS-PII.

Table S12. The C-index of AJCC system and nomograms.

Models	Training Cohort (*N* = 4154)		Validation Cohort (*N* = 5161)	
	C-index	95% CI	C-index	95% CI
Overall Survival				
AJCC system	0.654	0.639–0.669	0.700	0.678–0.722
Nomogram	0.718	0.704–0.731	0.765	0.745–0.785
Disease-free Survival				
AJCC system	0.654	0.638–0.671	0.657	0.640–0.674
Nomogram	0.700	0.684–0.716	0.698	0.681–0.715

Abbreviations: AJCC, American Joint Committee on Cancer.

Table S13. Time-dependent ROC analyses stratified by TNM staging for predicting survival of patients with colorectal cancer in the training cohort.

Marker	1-Year	2-Year	3-Year	4-Year	5-Year	6-Year	7-Year	8-Year	9-Year	10-Year	11-Year
Overall Survival											
NLR (overall)	0.581	0.549	0.542	0.531	0.524	0.525	0.525	0.514	0.494	0.462	0.460
NLR (stage II)	0.604	0.564	0.555	0.540	0.521	0.525	0.530	0.524	0.501	0.466	0.467
NLR (stage III)	0.588	0.561	0.558	0.548	0.549	0.549	0.552	0.524	0.531	0.523	0.502
PLR (overall)	0.578	0.532	0.519	0.511	0.502	0.479	0.469	0.463	0.455	0.430	0.372
PLR (stage II)	0.511	0.539	0.518	0.514	0.497	0.481	0.473	0.467	0.462	0.434	0.383
PLR (stage III)	0.615	0.543	0.533	0.520	0.515	0.478	0.464	0.466	0.453	0.449	0.261
LMR (overall)	0.587	0.553	0.543	0.537	0.529	0.535	0.536	0.520	0.511	0.510	0.552
LMR (stage II)	0.571	0.556	0.557	0.545	0.532	0.540	0.548	0.536	0.521	0.518	0.569
LMR (stage III)	0.607	0.567	0.553	0.551	0.542	0.548	0.528	0.487	0.535	0.565	0.489
SII (overall)	0.596	0.552	0.538	0.524	0.515	0.502	0.498	0.489	0.473	0.446	0.403
SII (stage II)	0.576	0.554	0.540	0.527	0.508	0.499	0.500	0.493	0.477	0.452	0.415
SII (stage III)	0.619	0.570	0.557	0.541	0.539	0.521	0.513	0.501	0.485	0.443	0.281
OS-PII (overall)	0.586	0.564	0.562	0.550	0.545	0.557	0.566	0.547	0.534	0.485	0.527
OS-PII (stage II)	0.587	0.561	0.588	0.555	0.544	0.558	0.575	0.561	0.551	0.503	0.549
OS-PII (stage III)	0.596	0.579	0.565	0.565	0.562	0.574	0.566	0.520	0.493	0.433	0.354
Disease-free Survival											
NLR (overall)	0.531	0.520	0.522	0.520	0.522	0.519	0.522	0.506	0.489	0.447	0.449
NLR (stage II)	0.513	0.523	0.528	0.529	0.530	0.525	0.533	0.519	0.497	0.457	0.458
NLR (stage III)	0.559	0.539	0.542	0.539	0.546	0.547	0.553	0.527	0.566	0.521	0.624
PLR (overall)	0.514	0.503	0.495	0.490	0.486	0.462	0.456	0.445	0.434	0.399	0.346
PLR (stage II)	0.481	0.517	0.511	0.517	0.509	0.485	0.479	0.464	0.451	0.415	0.367
PLR (stage III)	0.544	0.511	0.500	0.483	0.482	0.451	0.447	0.451	0.445	0.423	0.287
LMR (overall)	0.538	0.523	0.522	0.521	0.525	0.525	0.527	0.508	0.496	0.483	0.531
LMR (stage II)	0.530	0.531	0.538	0.542	0.547	0.545	0.551	0.535	0.511	0.499	0.551
LMR (stage III)	0.558	0.536	0.529	0.527	0.528	0.531	0.513	0.476	0.554	0.550	0.562
SII (overall)	0.530	0.518	0.515	0.508	0.506	0.491	0.490	0.480	0.464	0.425	0.385
SII (stage II)	0.510	0.520	0.526	0.527	0.519	0.501	0.505	0.496	0.478	0.443	0.406
SII (stage III)	0.557	0.538	0.530	0.517	0.521	0.507	0.504	0.495	0.502	0.429	0.318
DFS-PII (overall)	0.569	0.549	0.548	0.550	0.558	0.569	0.578	0.571	0.562	0.520	0.550
DFS-PII (stage II)	0.631	0.586	0.572	0.570	0.578	0.584	0.595	0.587	0.575	0.530	0.560
DFS-PII (stage III)	0.543	0.531	0.538	0.541	0.550	0.563	0.568	0.549	0.561	0.529	0.595

Abbreviations: TNM, Tumor-node-metastasis.

Table S14. Time-dependent ROC analyses stratified by TNM staging for predicting survival of patients with colorectal cancer in the validation cohort.

**Marker**	**1-Year**	**2-Year**	**3-Year**	**4-Year**	**5-Year**	**6-Year**	**7-Year**
Overall Survival							
NLR (overall)	0.661	0.623	0.592	0.592	0.600	0.612	0.669
NLR (stage II)	0.688	0.592	0.569	0.561	0.587	0.570	0.688
NLR (stage III)	0.663	0.635	0.603	0.606	0.607	0.631	0.668
PLR (overall)	0.589	0.554	0.550	0.520	0.528	0.532	0.608
PLR (stage II)	0.617	0.492	0.506	0.453	0.492	0.507	0.637
PLR (stage III)	0.591	0.571	0.563	0.541	0.543	0.543	0.596
LMR (overall)	0.658	0.603	0.560	0.539	0.528	0.549	0.638
LMR (stage II)	0.709	0.571	0.553	0.522	0.528	0.516	0.616
LMR (stage III)	0.657	0.618	0.569	0.552	0.537	0.575	0.660
SII (overall)	0.653	0.602	0.576	0.551	0.555	0.558	0.633
SII (stage II)	0.639	0.537	0.522	0.458	0.501	0.476	0.615
SII (stage III)	0.664	0.623	0.594	0.583	0.572	0.594	0.644
OS-PII (overall)	0.625	0.599	0.566	0.575	0.561	0.587	0.628
OS-PII (stage II)	0.712	0.638	0.601	0.618	0.609	0.623	0.622
OS-PII (stage III)	0.614	0.601	0.562	0.572	0.554	0.594	0.659
Disease-free Survival							
NLR (overall)	0.587	0.558	0.559	0.573	0.586	0.591	0.648
NLR (stage II)	0.554	0.535	0.546	0.542	0.559	0.544	0.632
NLR (stage III)	0.602	0.571	0.569	0.591	0.602	0.617	0.660
PLR (overall)	0.552	0.521	0.520	0.510	0.509	0.519	0.607
PLR (stage II)	0.525	0.495	0.493	0.464	0.471	0.507	0.627
PLR (stage III)	0.566	0.534	0.532	0.529	0.528	0.526	0.595
LMR (overall)	0.587	0.544	0.514	0.500	0.491	0.520	0.618
LMR (stage II)	0.557	0.508	0.494	0.475	0.468	0.482	0.580
LMR (stage III)	0.601	0.563	0.529	0.518	0.512	0.555	0.649
SII (overall)	0.577	0.540	0.536	0.534	0.537	0.541	0.620
SII (stage II)	0.520	0.490	0.497	0.474	0.488	0.479	0.579
SII (stage III)	0.599	0.561	0.554	0.561	0.557	0.574	0.643
DFS-PII (overall)	0.550	0.549	0.535	0.540	0.541	0.556	0.519
DFS-PII (stage II)	0.604	0.599	0.572	0.587	0.601	0.613	0.618
DFS-PII (stage III)	0.534	0.536	0.519	0.519	0.503	0.514	0.462

Abbreviations: TNM, Tumor-node-metastasis.

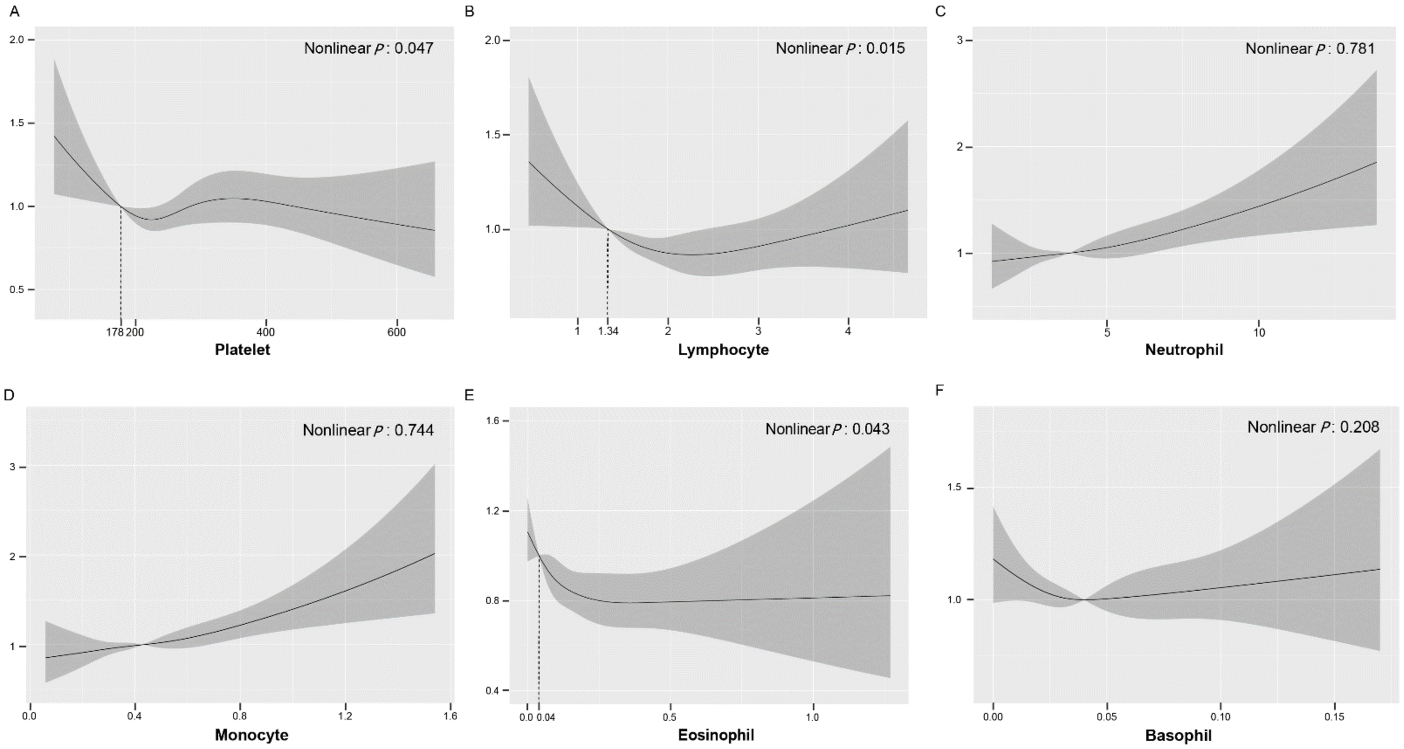

Figure S1. Restricted cubic spline regression for assessing the nonlinear relationship between preoperative peripheral blood cell counts and overall survival of patients with colorectal cancer.

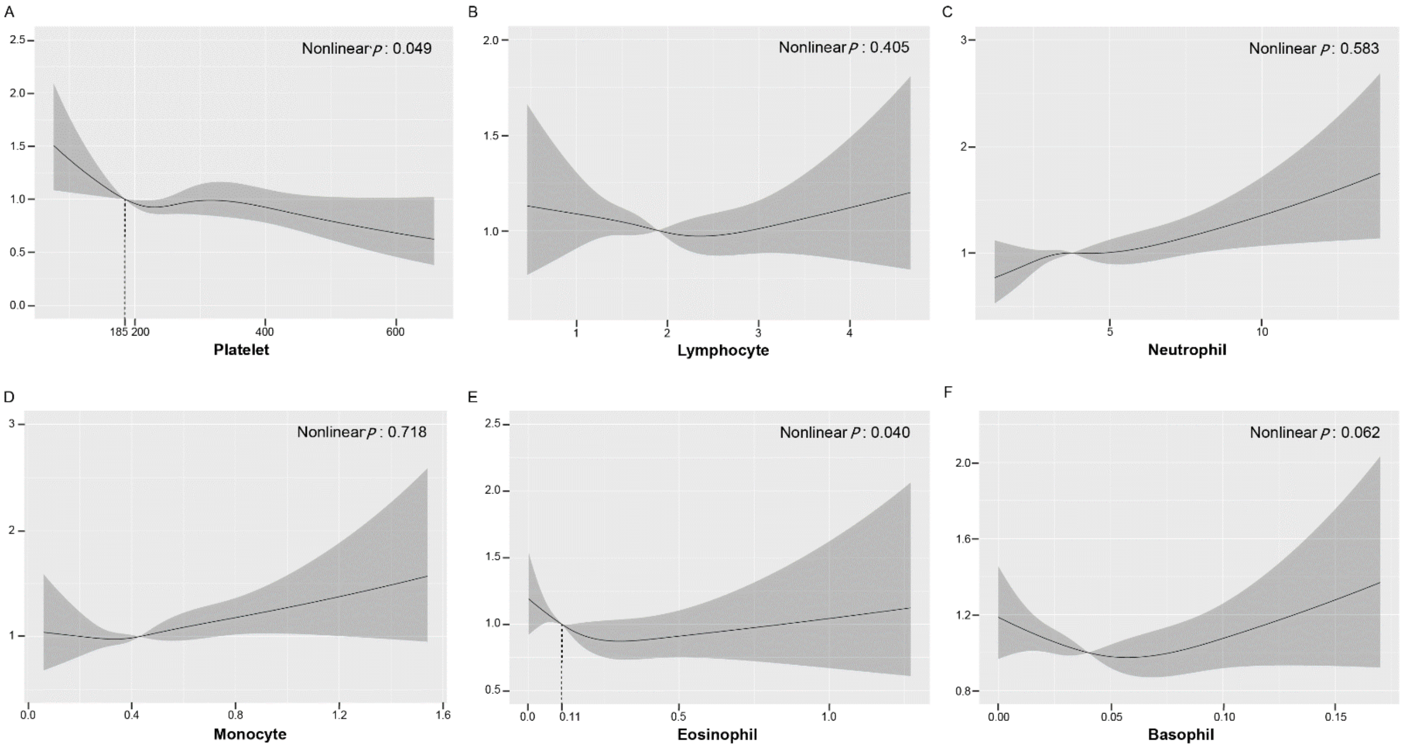

Figure S2. Restricted cubic spline regression for assessing the nonlinear relationship between preoperative peripheral blood cell counts and disease-free survival of patients with colorectal cancer.

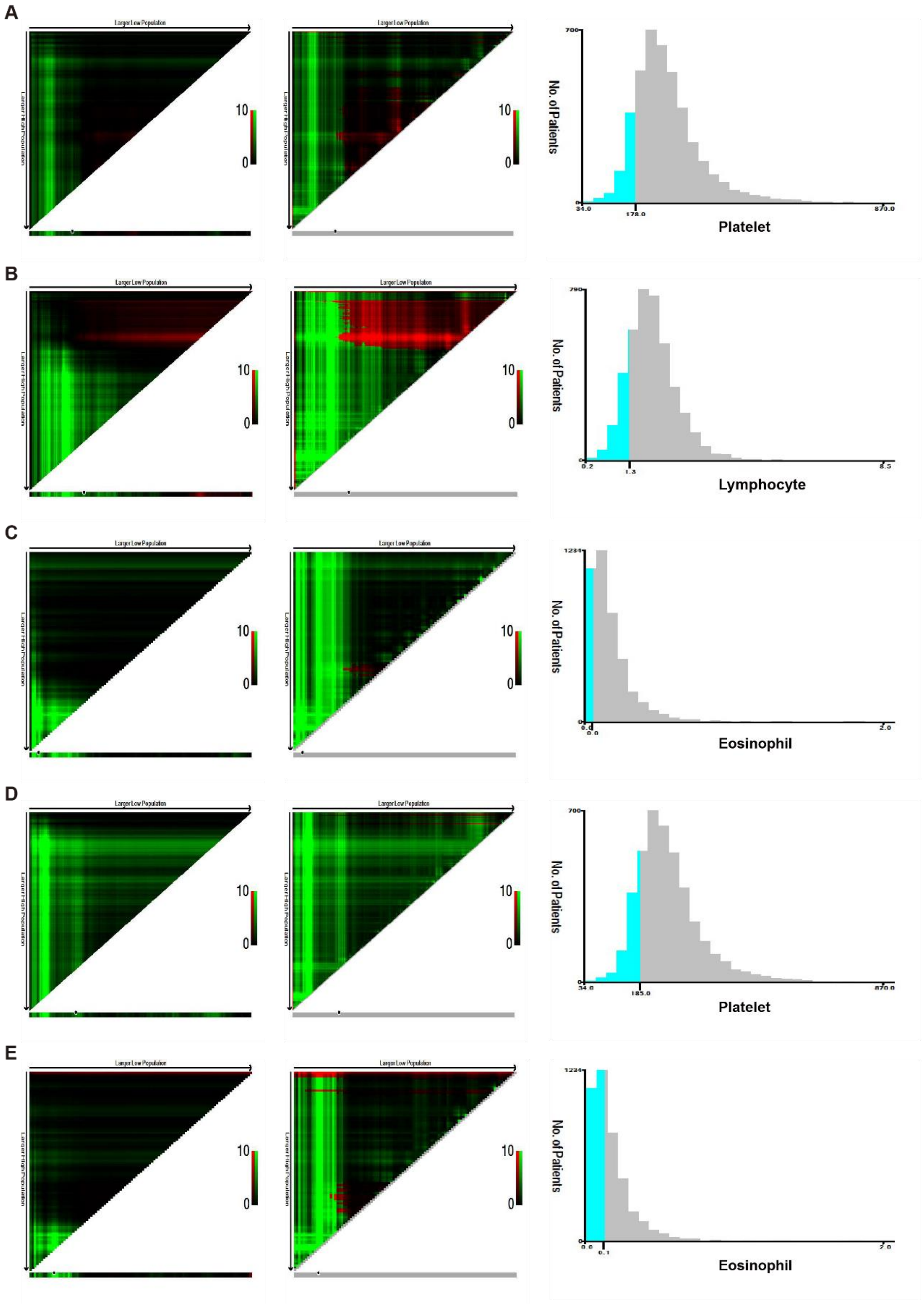

Figure S3. Determination of the cut-off value for platelet, lymphocyte, and eosinophil counts. X-tile analyses of overall survival (**A**–**C**) and disease-free survival (**D**–**E**) were performed using patients' data to determine the cut-off value for platelet, lymphocyte and eosinophil counts.

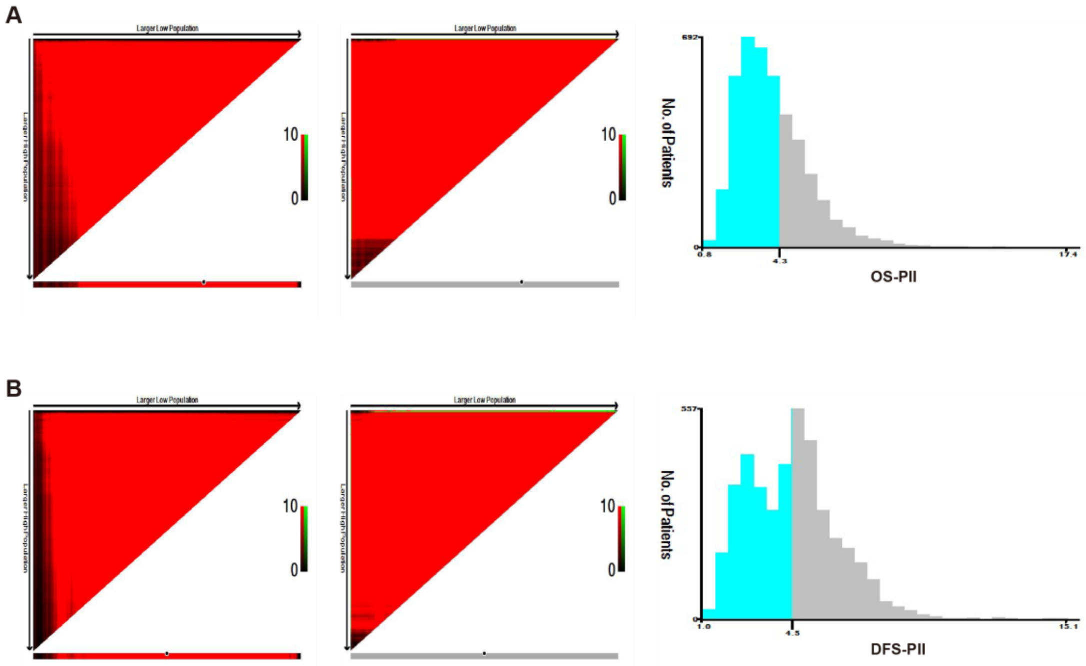

Figure S4. Determination of the optimal cut-off value for the OS-PII and DFS-PII. X-tile analyses were performed using patients' data to determine the optimal cut-off value for the OS-PII (**A**) and DFS-PII (**B**).

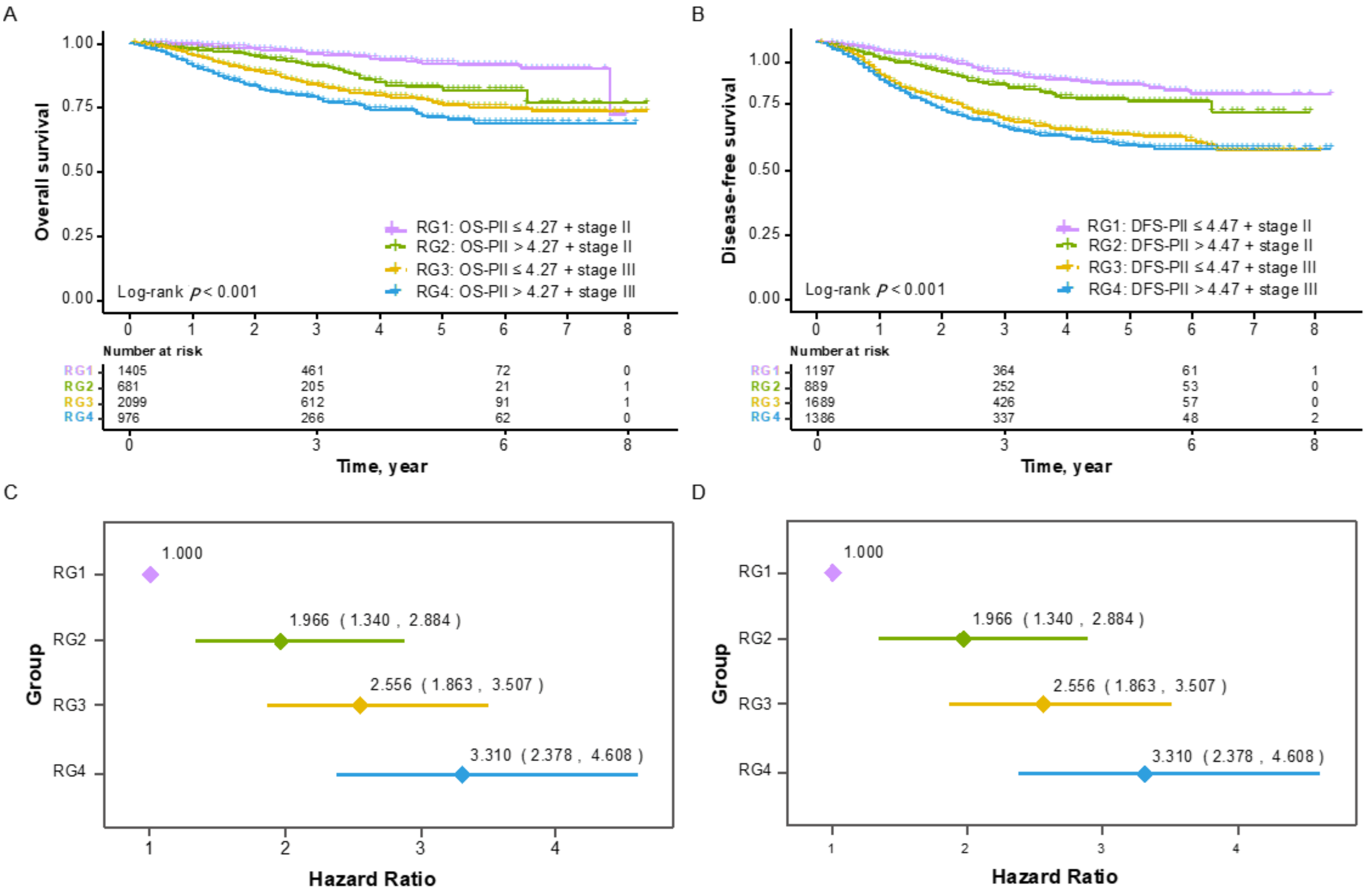

Figure S5. Risk stratification combining PIIs and TNM staging in relation to overall survival and disease-free survival of colorectal cancer in the validation cohort. Kaplan-Meier curves of four risk groups for overall survival (**A**) and disease-free survival (**B**). Multivariate Cox analyses of the four risk groups for overall survival (**C**) and disease-free survival (**D**) adjusting for the significant clinicopathological factors in relation to overall survival (Table 4) and disease-free survival (Table 5). Abbreviations: TNM, Tumor-node-metastasis.

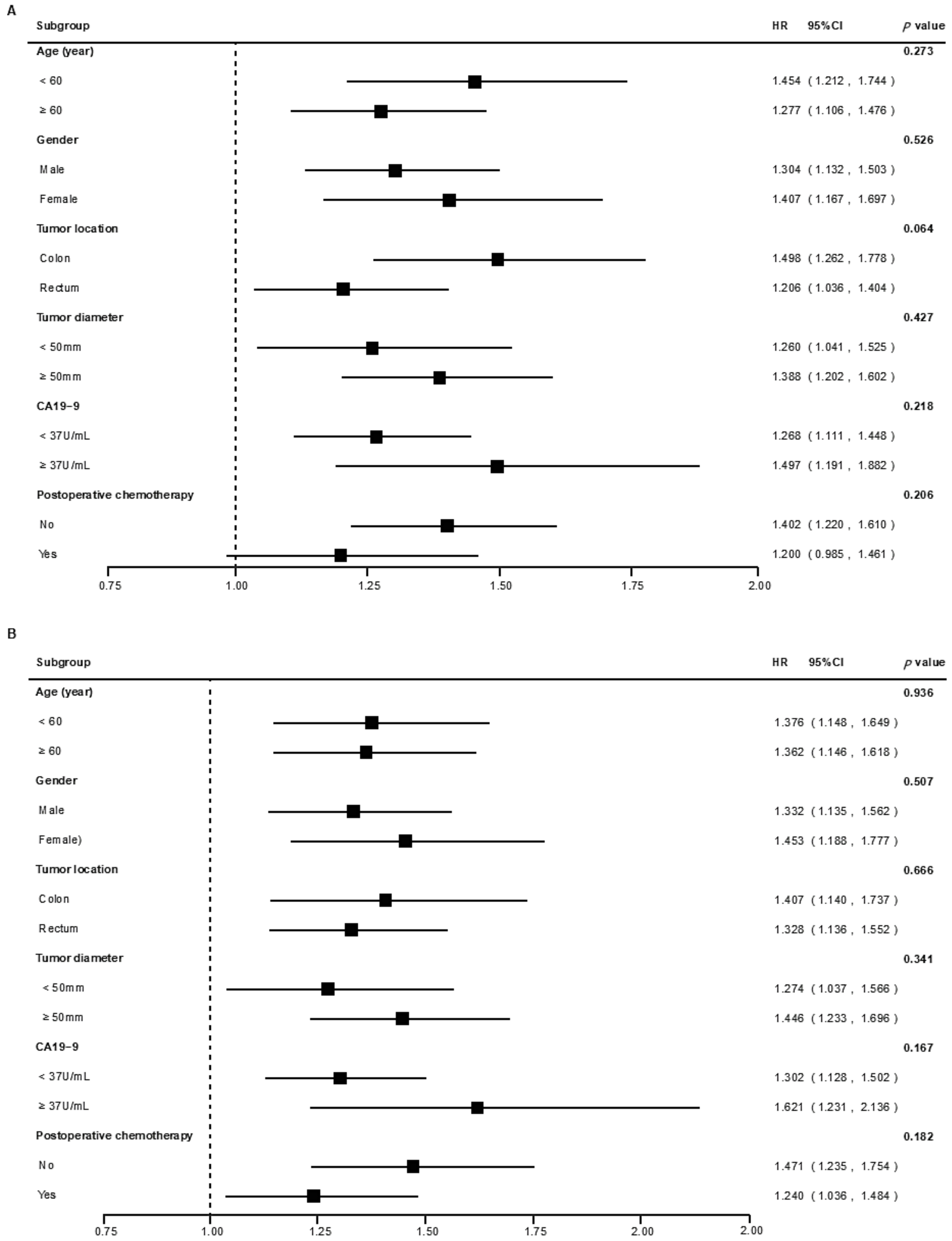

Figure S6. Subgroup analyses for the association of OS-PII and DFS-PII with the prognosis of colorectal cancer in the training cohort. Subgroup analyses were performed in colorectal cancer patients stratified by age, gender, tumor location, tumor diameter, CA19-9, and postoperative chemotherapy (no or yes) to assess the association between OS-PII and overall survival (**A**); and the association between DFS-PII and disease-free survival (**B**). All the analyses were adjusted for the significant clinicopathological factors in relation to overall survival (Table 2) and disease-free survival (Table 3). Abbreviations: HR, hazard ratio; CI, confidence interval.

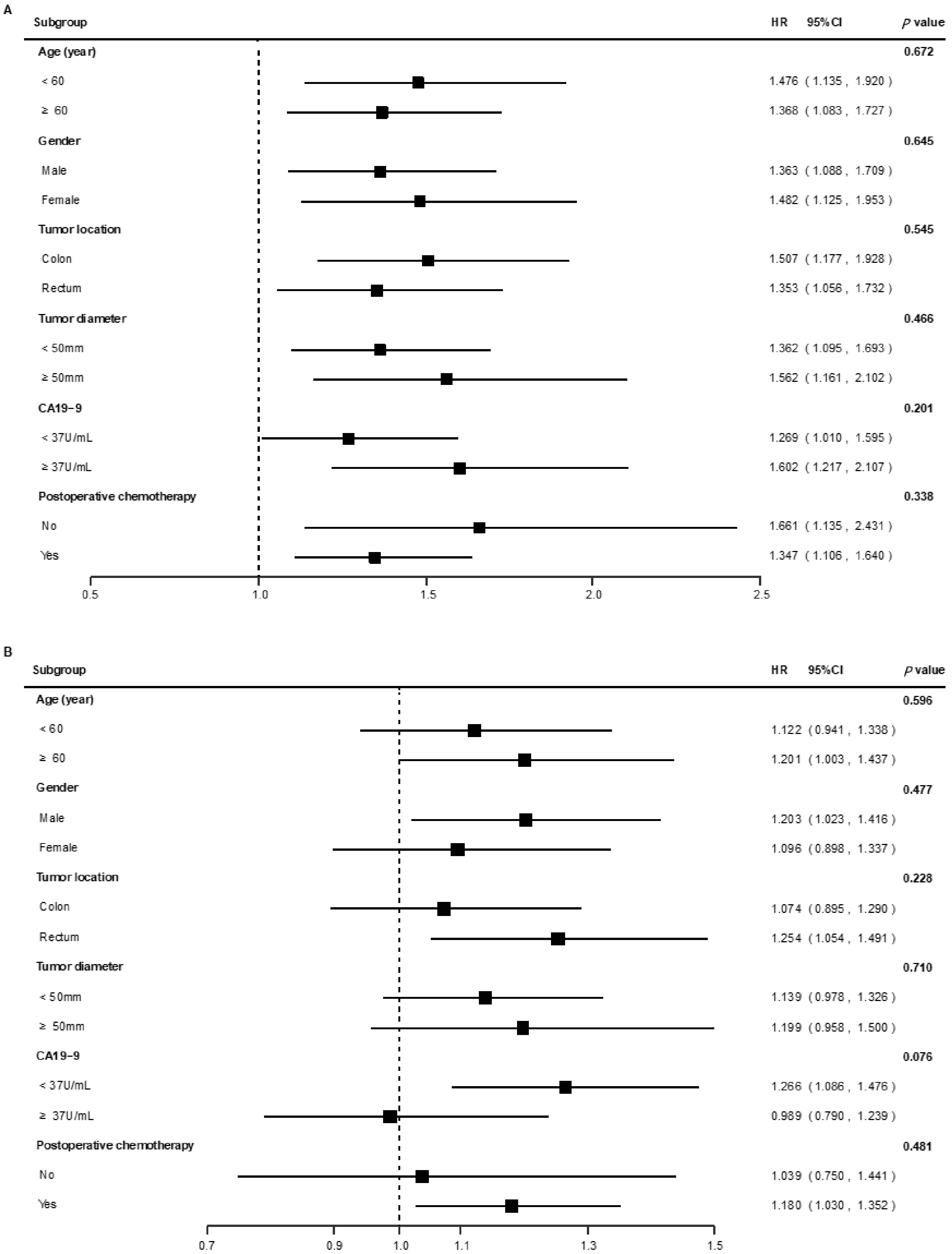

Figure S7. Subgroup analyses for the association of OS-PII and DFS-PII with the prognosis of colorectal cancer in the validation cohort. Subgroup analyses were performed in colorectal cancer patients stratified by age, gender, tumor location, tumor diameter, CA19-9, and postoperative chemotherapy (no or yes) to assess the association between OS-PII and overall survival (**A**); and the association between DFS-PII and disease-free survival (**B**). All the analyses were adjusted for the significant clinicopathological factors in relation to overall survival (Table 4) and disease-free survival (Table 5). Abbreviations: HR, hazard ratio; CI, confidence interval.

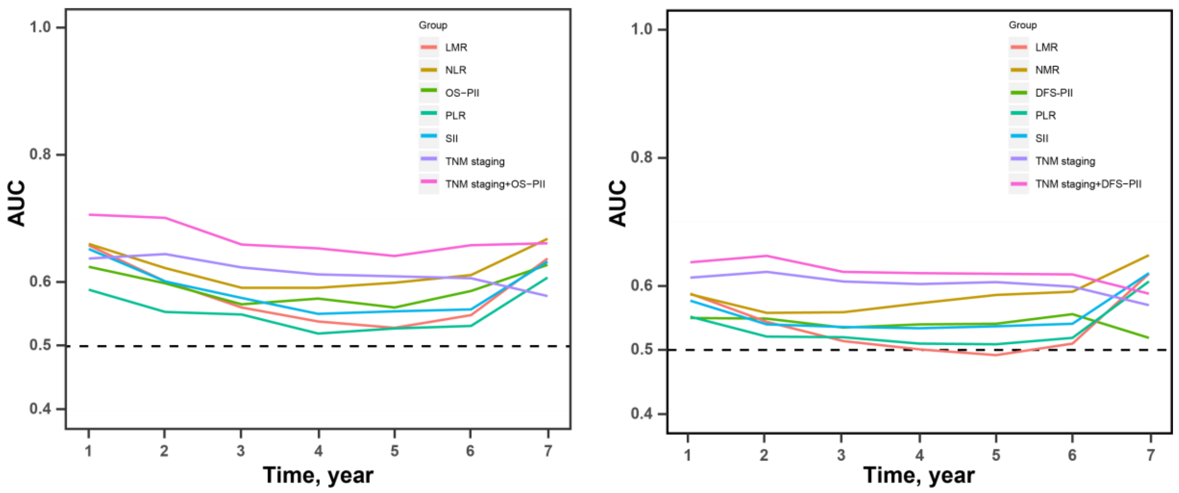

Figure S8. The time-dependent AUCs of PIIs, TNM staging, a combination of PIIs and TNM staging, NLR, PLR, LMR, and SII in the training cohort. Time-dependent AUCs presented the sequential trends of PIIs, TNM staging, a model of PIIs and TNM staging, NLR, PLR, LMR, and SII for overall survival prediction (**A**) and disease-free survival prediction (**B**). The horizontal axis represents the years after radical resection, and the vertical axis represents the estimated area under the ROC curves for survival at the time of interest. Abbreviations: TNM, Tumor-node-metastasis.

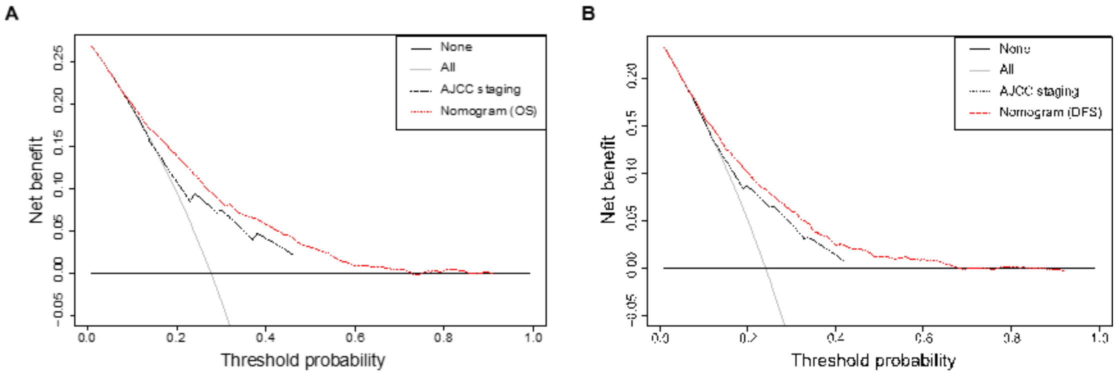

Figure S9. Decision curve analysis for 5-year overall survival prediction **(A)** and 5-year disease-free survival prediction **(B)** in the training cohort. Abbreviations: AJCC, American Joint Committee on Cancer.

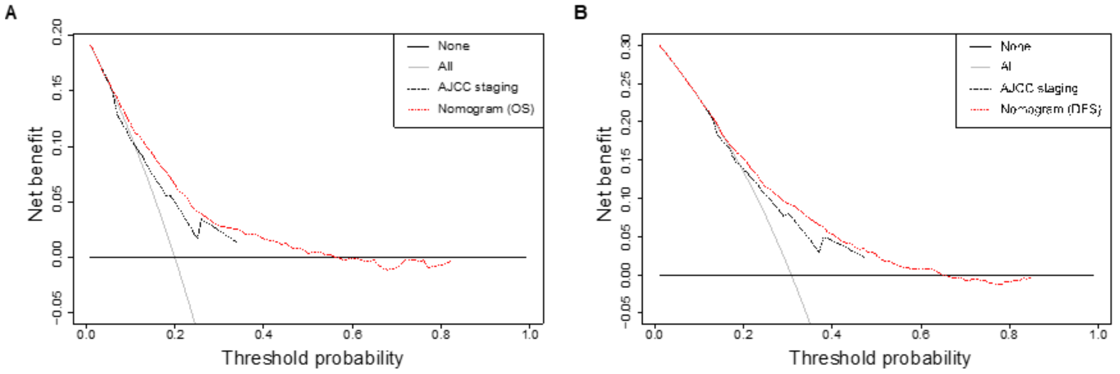

Figure S10. Decision curve analysis for 5-year overall survival prediction (**A**) and 5-year disease-free survival prediction (**B**) in the validation cohort. Abbreviations: AJCC, American Joint Committee on Cancer.
